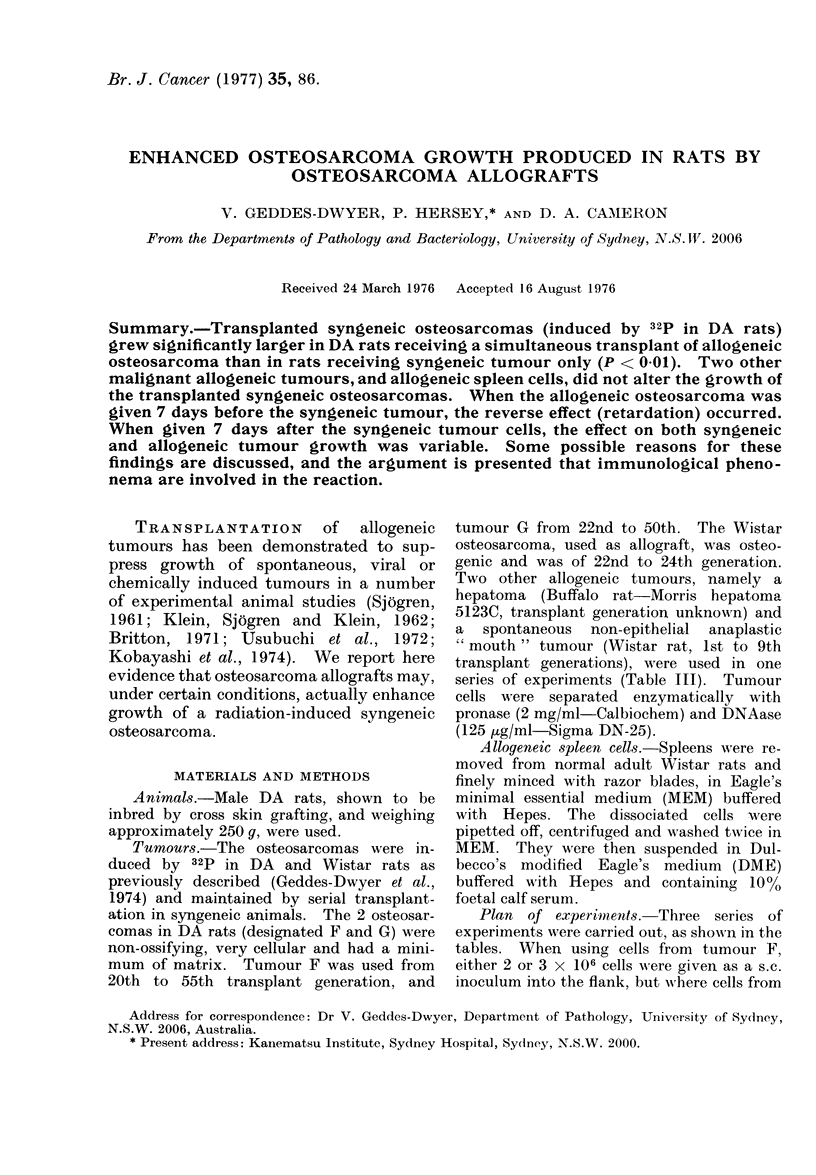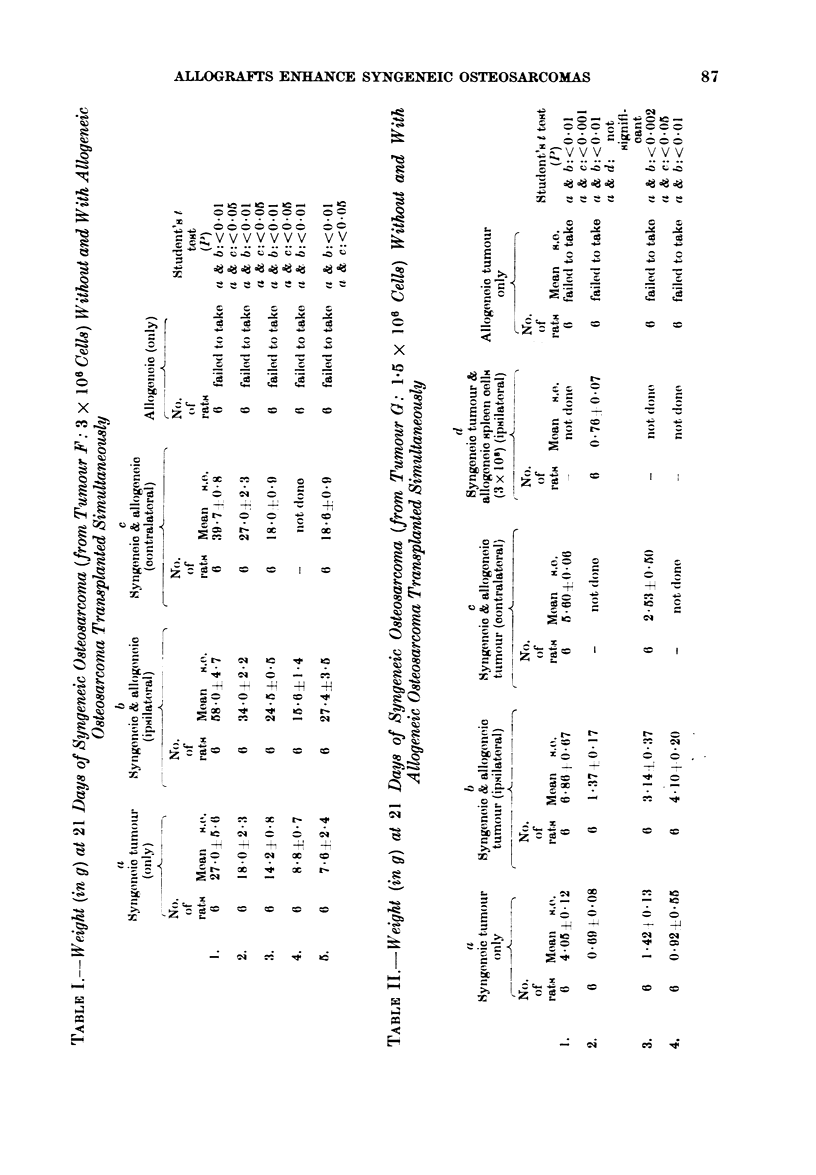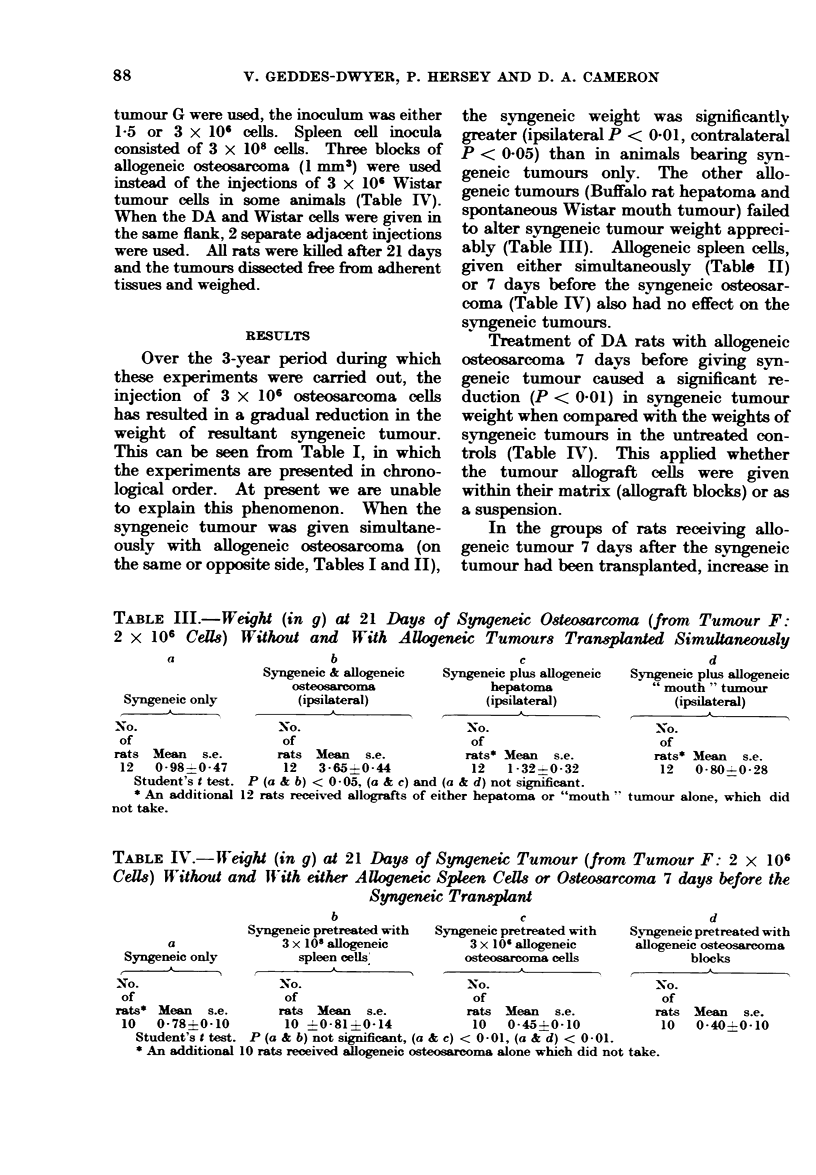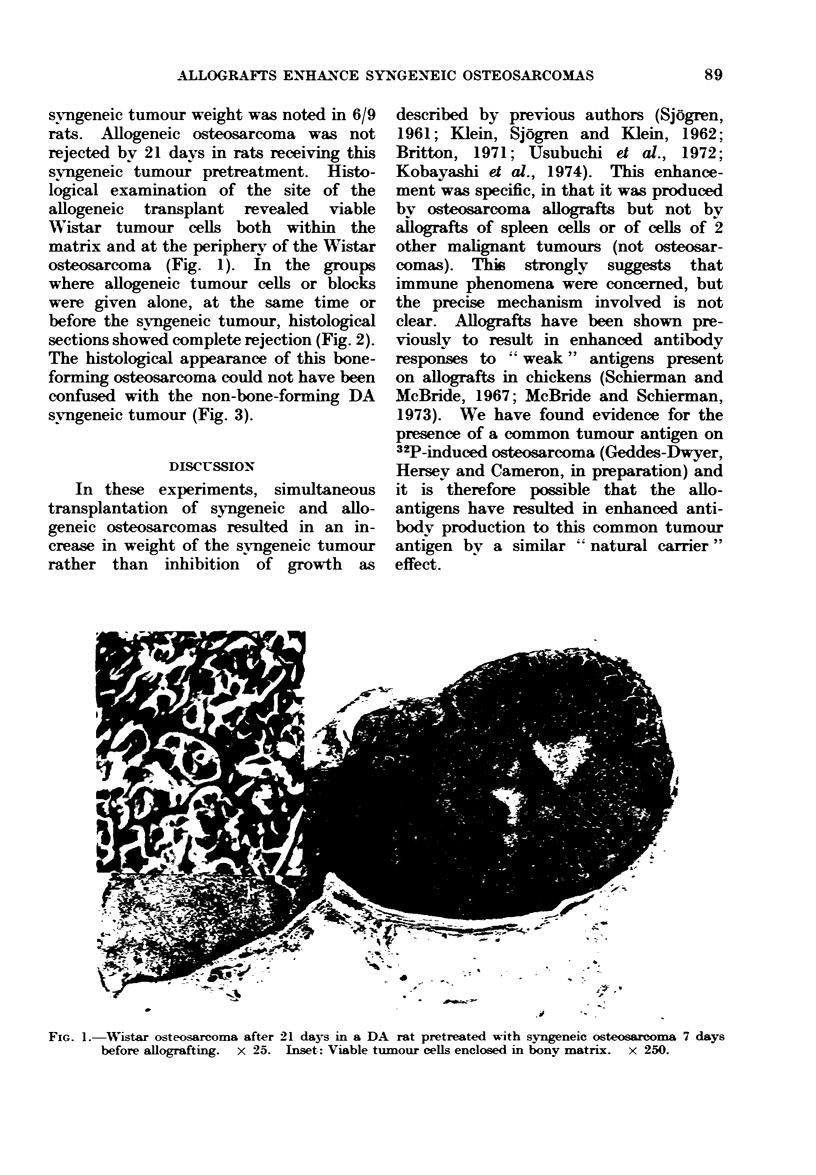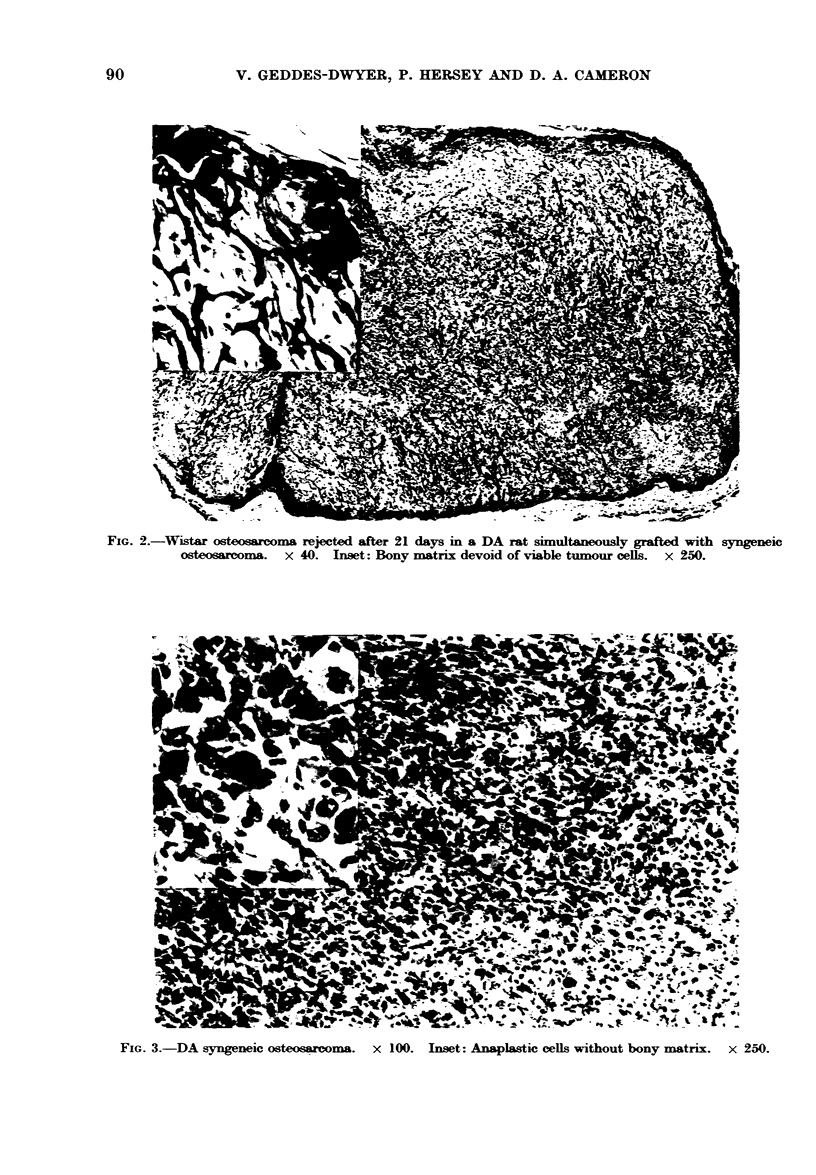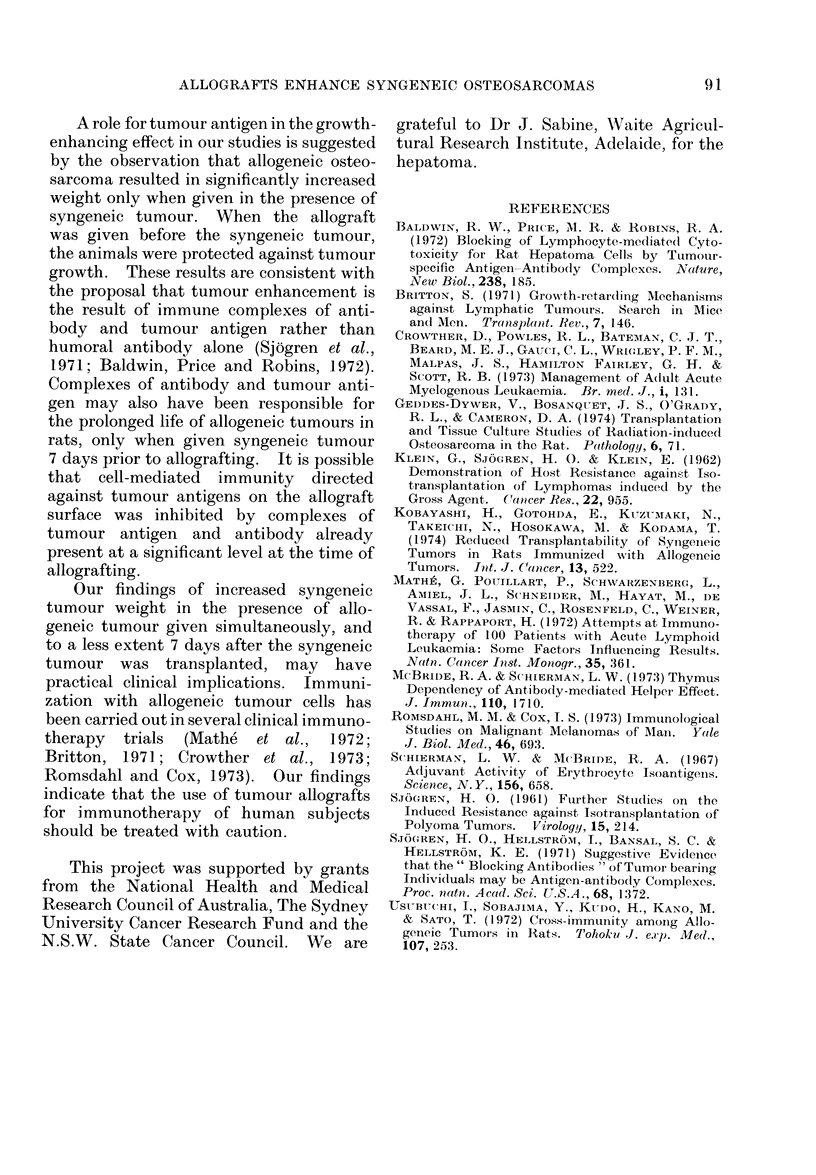# Enhanced osteosarcoma growth produced in rats by osteosarcoma allografts.

**DOI:** 10.1038/bjc.1977.7

**Published:** 1977-01

**Authors:** V. Geddes-Dwyer, P. Hersey, D. A. Cameron

## Abstract

**Images:**


*Br. J. Cancer* (1977) **35**, 86.

### ENHANCED OSTEOSARCOMA GROWTH PRODUCED IN RATS BY OSTEOSARCOMA ALLOGRAFTS

V. GEDDES-DWYER, P. HERSEY,* AND D. A. CAMERON

*From the Departments of Pathology and Bacteriology, University of Sydney, N.S.W. 2006*

Received 24 March 1976 Accepted 16 August 1976

**Summary.—Transplanted syngeneic osteosarcomas (induced by $^{32}P$ in DA rats) grew significantly larger in DA rats receiving a simultaneous transplant of allogeneic osteosarcoma than in rats receiving syngeneic tumour only ($P < 0 \cdot 01$). Two other malignant allogeneic tumours, and allogeneic spleen cells, did not alter the growth of the transplanted syngeneic osteosarcomas. When the allogeneic osteosarcoma was given 7 days before the syngeneic tumour, the reverse effect (retardation) occurred. When given 7 days after the syngeneic tumour cells, the effect on both syngeneic and allogeneic tumour growth was variable. Some possible reasons for these findings are discussed, and the argument is presented that immunological phenomena are involved in the reaction.**

TRANSPLANTATION of allogeneic tumours has been demonstrated to suppress growth of spontaneous, viral or chemically induced tumours in a number of experimental animal studies (Sjögren, 1961; Klein, Sjögren and Klein, 1962; Britton, 1971; Usubuchi *et al.*, 1972; Kobayashi *et al.*, 1974). We report here evidence that osteosarcoma allografts may, under certain conditions, actually enhance growth of a radiation-induced syngeneic osteosarcoma.

### MATERIALS AND METHODS

*Animals.*—Male DA rats, shown to be inbred by cross skin grafting, and weighing approximately 250 *g*, were used.

*Tumours.*—The osteosarcomas were induced by $^{32}P$ in DA and Wistar rats as previously described (Geddes-Dwyer *et al.*, 1974) and maintained by serial transplantation in syngeneic animals. The 2 osteosarcomas in DA rats (designated F and G) were non-ossifying, very cellular and had a minimum of matrix. Tumour F was used from 20th to 55th transplant generation, and tumour G from 22nd to 50th. The Wistar osteosarcoma, used as allograft, was osteogenic and was of 22nd to 24th generation. Two other allogeneic tumours, namely a hepatoma (Buffalo rat—Morris hepatoma 5123C, transplant generation unknown) and a spontaneous non-epithelial anaplastic " mouth " tumour (Wistar rat, 1st to 9th transplant generations), were used in one series of experiments (Table III). Tumour cells were separated enzymatically with pronase (2 mg/ml—Calbiochem) and DNAase (125 $\mu$g/ml—Sigma DN-25).

*Allogeneic spleen cells.*—Spleens were removed from normal adult Wistar rats and finely minced with razor blades, in Eagle's minimal essential medium (MEM) buffered with Hepes. The dissociated cells were pipetted off, centrifuged and washed twice in MEM. They were then suspended in Dulbecco's modified Eagle's medium (DME) buffered with Hepes and containing 10% foetal calf serum.

*Plan of experiments.*—Three series of experiments were carried out, as shown in the tables. When using cells from tumour F, either 2 or 3 $\times 10^6$ cells were given as a s.c. inoculum into the flank, but where cells from

Address for correspondence: Dr V. Geddes-Dwyer, Department of Pathology, University of Sydney, N.S.W. 2006, Australia.

* Present address: Kanematsu Institute, Sydney Hospital, Sydney, N.S.W. 2000.

TABLE I.—*Weight (in g) at 21 Days of Syngeneic Osteosarcoma (from Tumour F: 3 × 10⁶ Cells) Without and With Allogeneic Osteosarcoma Transplanted Simultaneously*

| | *a* Syngeneic tumour (only) | | *b* Syngeneic & allogeneic (ipsilateral) | | *c* Syngeneic & allogeneic (contralateral) | | Allogeneic (only) | | Student's *t* test (*P*) |
|---|---|---|---|---|---|---|---|---|---|
| | No. of rats | Mean s.e. | No. of rats | Mean s.e. | No. of rats | Mean s.e. | No. of rats | | |
| 1. | 6 | 27·0±5·6 | 6 | 58·0±4·7 | 6 | 39·7±0·8 | 6 | failed to take | *a* & *b*:<0·01<br>*a* & *c*:<0·05 |
| 2. | 6 | 18·0±2·3 | 6 | 34·0±2·2 | 6 | 27·0±2·3 | 6 | failed to take | *a* & *b*:<0·01<br>*a* & *c*:<0·05 |
| 3. | 6 | 14·2±0·8 | 6 | 24·5±0·5 | 6 | 18·0±0·9 | 6 | failed to take | *a* & *b*:<0·01<br>*a* & *c*:<0·05 |
| 4. | 6 | 8·8±0·7 | 6 | 15·6±1·4 | – | not done | 6 | failed to take | *a* & *b*:<0·01 |
| 5. | 6 | 7·6±2·4 | 6 | 27·4±3·5 | 6 | 18·6±0·9 | 6 | failed to take | *a* & *b*:<0·01<br>*a* & *c*:<0·05 |

TABLE II.—*Weight (in g) at 21 Days of Syngeneic Osteosarcoma (from Tumour G: 1·5 × 10⁶ Cells) Without and With Allogeneic Osteosarcoma Transplanted Simultaneously*

| | *a* Syngeneic tumour only | | *b* Syngeneic & allogeneic tumour (ipsilateral) | | *c* Syngeneic & allogeneic tumour (contralateral) | | *d* Syngeneic tumour & allogeneic spleen cells (3 × 10⁸) (ipsilateral) | | Allogeneic tumour only | | Student's *t* test (*P*) |
|---|---|---|---|---|---|---|---|---|---|---|---|
| | No. of rats | Mean s.e. | No. of rats | Mean s.e. | No. of rats | Mean s.e. | No. of rats | Mean s.e. | No. of rats | Mean s.e. | |
| 1. | 6 | 4·05±0·12 | 6 | 6·86±0·67 | 6 | 5·60±0·06 | – | not done | 6 | failed to take | *a* & *b*:<0·01<br>*a* & *c*:<0·001 |
| 2. | 6 | 0·69±0·08 | 6 | 1·37±0·17 | – | not done | 6 | 0·76±0·07 | 6 | failed to take | *a* & *b*:<0·01<br>*a* & *d*: not significant |
| 3. | 6 | 1·42±0·13 | 6 | 3·14±0·37 | 6 | 2·53±0·50 | – | not done | 6 | failed to take | *a* & *b*:<0·002<br>*a* & *c*:<0·05 |
| 4. | 6 | 0·92±0·55 | 6 | 4·10±0·20 | – | not done | – | not done | 6 | failed to take | *a* & *b*:<0·01 |

tumour G were used, the inoculum was either 1·5 or 3 × 10[6] cells. Spleen cell inocula consisted of 3 × 10[8] cells. Three blocks of allogeneic osteosarcoma (1 mm³) were used instead of the injections of 3 × 10[6] Wistar tumour cells in some animals (Table IV). When the DA and Wistar cells were given in the same flank, 2 separate adjacent injections were used. All rats were killed after 21 days and the tumours dissected free from adherent tissues and weighed.

### RESULTS

Over the 3-year period during which these experiments were carried out, the injection of 3 × 10[6] osteosarcoma cells has resulted in a gradual reduction in the weight of resultant syngeneic tumour. This can be seen from Table I, in which the experiments are presented in chronological order. At present we are unable to explain this phenomenon. When the syngeneic tumour was given simultaneously with allogeneic osteosarcoma (on the same or opposite side, Tables I and II), the syngeneic weight was significantly greater (ipsilateral $P < 0·01$, contralateral $P < 0·05$) than in animals bearing syngeneic tumours only. The other allogeneic tumours (Buffalo rat hepatoma and spontaneous Wistar mouth tumour) failed to alter syngeneic tumour weight appreciably (Table III). Allogeneic spleen cells, given either simultaneously (Table II) or 7 days before the syngeneic osteosarcoma (Table IV) also had no effect on the syngeneic tumours.

Treatment of DA rats with allogeneic osteosarcoma 7 days before giving syngeneic tumour caused a significant reduction ($P < 0·01$) in syngeneic tumour weight when compared with the weights of syngeneic tumours in the untreated controls (Table IV). This applied whether the tumour allograft cells were given within their matrix (allograft blocks) or as a suspension.

In the groups of rats receiving allogeneic tumour 7 days after the syngeneic tumour had been transplanted, increase in

TABLE III.—*Weight (in g) at 21 Days of Syngeneic Osteosarcoma (from Tumour F: 2 × 10⁶ Cells) Without and With Allogeneic Tumours Transplanted Simultaneously*

| *a* Syngeneic only | | *b* Syngeneic & allogeneic osteosarcoma (ipsilateral) | | *c* Syngeneic plus allogeneic hepatoma (ipsilateral) | | *d* Syngeneic plus allogeneic "mouth" tumour (ipsilateral) | |
|---|---|---|---|---|---|---|---|
| No. of rats | Mean s.e. | No. of rats | Mean s.e. | No. of rats* | Mean s.e. | No. of rats* | Mean s.e. |
| 12 | 0·98±0·47 | 12 | 3·65±0·44 | 12 | 1·32±0·32 | 12 | 0·80±0·28 |

Student's *t* test. *P* (*a* & *b*) < 0·05, (*a* & *c*) and (*a* & *d*) not significant.

* An additional 12 rats received allografts of either hepatoma or "mouth" tumour alone, which did not take.

TABLE IV.—*Weight (in g) at 21 Days of Syngeneic Tumour (from Tumour F: 2 × 10⁶ Cells) Without and With either Allogeneic Spleen Cells or Osteosarcoma 7 days before the Syngeneic Transplant*

| *a* Syngeneic only | | *b* Syngeneic pretreated with 3 × 10⁸ allogeneic spleen cells | | *c* Syngeneic pretreated with 3 × 10⁶ allogeneic osteosarcoma cells | | *d* Syngeneic pretreated with allogeneic osteosarcoma blocks | |
|---|---|---|---|---|---|---|---|
| No. of rats* | Mean s.e. | No. of rats | Mean s.e. | No. of rats | Mean s.e. | No. of rats* | Mean s.e. |
| 10 | 0·78±0·10 | 10 | ±0·81±0·14 | 10 | 0·45±0·10 | 10 | 0·40±0·10 |

Student's *t* test. *P* (*a* & *b*) not significant, (*a* & *c*) < 0·01, (*a* & *d*) < 0·01.

* An additional 10 rats received allogeneic osteosarcoma alone which did not take.

syngeneic tumour weight was noted in 6/9 rats. Allogeneic osteosarcoma was not rejected by 21 days in rats receiving this syngeneic tumour pretreatment. Histological examination of the site of the allogeneic transplant revealed viable Wistar tumour cells both within the matrix and at the periphery of the Wistar osteosarcoma (Fig. 1). In the groups where allogeneic tumour cells or blocks were given alone, at the same time or before the syngeneic tumour, histological sections showed complete rejection (Fig. 2). The histological appearance of this bone-forming osteosarcoma could not have been confused with the non-bone-forming DA syngeneic tumour (Fig. 3).

### DISCUSSION

In these experiments, simultaneous transplantation of syngeneic and allogeneic osteosarcomas resulted in an increase in weight of the syngeneic tumour rather than inhibition of growth as described by previous authors (Sjögren, 1961; Klein, Sjögren and Klein, 1962; Britton, 1971; Usubuchi *et al.*, 1972; Kobayashi *et al.*, 1974). This enhancement was specific, in that it was produced by osteosarcoma allografts but not by allografts of spleen cells or of cells of 2 other malignant tumours (not osteosarcomas). This strongly suggests that immune phenomena were concerned, but the precise mechanism involved is not clear. Allografts have been shown previously to result in enhanced antibody responses to " weak " antigens present on allografts in chickens (Schierman and McBride, 1967; McBride and Schierman, 1973). We have found evidence for the presence of a common tumour antigen on $^{32}$P-induced osteosarcoma (Geddes-Dwyer, Hersey and Cameron, in preparation) and it is therefore possible that the alloantigens have resulted in enhanced antibody production to this common tumour antigen by a similar " natural carrier " effect.

FIG. 1.—Wistar osteosarcoma after 21 days in a DA rat pretreated with syngeneic osteosarcoma 7 days before allografting. × 25. Inset: Viable tumour cells enclosed in bony matrix. × 250.

FIG. 2.—Wistar osteosarcoma rejected after 21 days in a DA rat simultaneously grafted with syngeneic osteosarcoma. × 40. Inset: Bony matrix devoid of viable tumour cells. × 250.

FIG. 3.—DA syngeneic osteosarcoma. × 100. Inset: Anaplastic cells without bony matrix. × 250.

A role for tumour antigen in the growth-enhancing effect in our studies is suggested by the observation that allogeneic osteosarcoma resulted in significantly increased weight only when given in the presence of syngeneic tumour. When the allograft was given before the syngeneic tumour, the animals were protected against tumour growth. These results are consistent with the proposal that tumour enhancement is the result of immune complexes of antibody and tumour antigen rather than humoral antibody alone (Sjögren *et al.*, 1971; Baldwin, Price and Robins, 1972). Complexes of antibody and tumour antigen may also have been responsible for the prolonged life of allogeneic tumours in rats, only when given syngeneic tumour 7 days prior to allografting. It is possible that cell-mediated immunity directed against tumour antigens on the allograft surface was inhibited by complexes of tumour antigen and antibody already present at a significant level at the time of allografting.

Our findings of increased syngeneic tumour weight in the presence of allogeneic tumour given simultaneously, and to a less extent 7 days after the syngeneic tumour was transplanted, may have practical clinical implications. Immunization with allogeneic tumour cells has been carried out in several clinical immunotherapy trials (Mathé *et al.*, 1972; Britton, 1971; Crowther *et al.*, 1973; Romsdahl and Cox, 1973). Our findings indicate that the use of tumour allografts for immunotherapy of human subjects should be treated with caution.

This project was supported by grants from the National Health and Medical Research Council of Australia, The Sydney University Cancer Research Fund and the N.S.W. State Cancer Council. We are grateful to Dr J. Sabine, Waite Agricultural Research Institute, Adelaide, for the hepatoma.

### REFERENCES

BALDWIN, R. W., PRICE, M. R. & ROBINS, R. A. (1972) Blocking of Lymphocyte-mediated Cytotoxicity for Rat Hepatoma Cells by Tumour-specific Antigen–Antibody Complexes. *Nature, New Biol.*, **238**, 185.

BRITTON, S. (1971) Growth-retarding Mechanisms against Lymphatic Tumours. Search in Mice and Men. *Transplant. Rev.*, **7**, 146.

CROWTHER, D., POWLES, R. L., BATEMAN, C. J. T., BEARD, M. E. J., GAUCI, C. L., WRIGLEY, P. F. M., MALPAS, J. S., HAMILTON FAIRLEY, G. H. & SCOTT, R. B. (1973) Management of Adult Acute Myelogenous Leukaemia. *Br. med. J.*, **i**, 131.

GEDDES-DYWER, V., BOSANQUET, J. S., O'GRADY, R. L., & CAMERON, D. A. (1974) Transplantation and Tissue Culture Studies of Radiation-induced Osteosarcoma in the Rat. *Pathology*, **6**, 71.

KLEIN, G., SJÖGREN, H. O. & KLEIN, E. (1962) Demonstration of Host Resistance against Isotransplantation of Lymphomas induced by the Gross Agent. *Cancer Res.*, **22**, 955.

KOBAYASHI, H., GOTOHDA, E., KUZUMAKI, N., TAKEICHI, N., HOSOKAWA, M. & KODAMA, T. (1974) Reduced Transplantability of Syngeneic Tumors in Rats Immunized with Allogeneic Tumors. *Int. J. Cancer*, **13**, 522.

MATHÉ, G. POUILLART, P., SCHWARZENBERG, L., AMIEL, J. L., SCHNEIDER, M., HAYAT, M., DE VASSAL, F., JASMIN, C., ROSENFELD, C., WEINER, R. & RAPPAPORT, H. (1972) Attempts at Immunotherapy of 100 Patients with Acute Lymphoid Leukaemia: Some Factors Influencing Results. *Natn. Cancer Inst. Monogr.*, **35**, 361.

MCBRIDE, R. A. & SCHIERMAN, L. W. (1973) Thymus Dependency of Antibody-mediated Helper Effect. *J. Immun.*, **110**, 1710.

ROMSDAHL, M. M. & COX, I. S. (1973) Immunological Studies on Malignant Melanomas of Man. *Yale J. Biol. Med.*, **46**, 693.

SCHIERMAN, L. W. & MCBRIDE, R. A. (1967) Adjuvant Activity of Erythrocyte Isoantigens. *Science, N.Y.*, **156**, 658.

SJÖGREN, H. O. (1961) Further Studies on the Induced Resistance against Isotransplantation of Polyoma Tumors. *Virology*, **15**, 214.

SJÖGREN, H. O., HELLSTRÖM, I., BANSAL, S. C. & HELLSTRÖM, K. E. (1971) Suggestive Evidence that the " Blocking Antibodies " of Tumor bearing Individuals may be Antigen-antibody Complexes. *Proc. natn. Acad. Sci. U.S.A.*, **68**, 1372.

USUBUCHI, I., SOBAJIMA, Y., KUDO, H., KANO, M. & SATO, T. (1972) Cross-immunity among Allogeneic Tumors in Rats. *Tohoku J. exp. Med.*, **107**, 253.